# Acute and chronic ocular outcomes in SJS/TEN patients treated with oral ciclosporin vs intravenous immunoglobulin

**DOI:** 10.3389/fmed.2024.1398506

**Published:** 2024-08-19

**Authors:** Valencia Hui Xian Foo, Lee Haur Yueh, Jodhbir S. Mehta, Hon Shing Ong

**Affiliations:** ^1^Corneal and External Diseases Department, Singapore National Eye Centre, Singapore, Singapore; ^2^Tissue Engineering and Cell Therapy Department, Singapore Eye Research Institute, Singapore, Singapore; ^3^Department of Dermatology, Singapore General Hospital, Singapore, Singapore; ^4^Ophthalmology and Visual Science Academic Clinical Research Program, Duke-NUS Medical School, Singapore, Singapore; ^5^School of Material Science & Engineering and School of Mechanical and Aerospace Engineering, Nanyang Technological University, Singapore, Singapore

**Keywords:** ocular, Stevens-Johnson syndrome, toxic epidermal necrolysis, Ciclosporin, intravenous immunoglobulin

## Abstract

**Background/Aim:**

To evaluate differences in ocular complications of Stevens Johnson Syndrome (SJS)/Toxic Epidermal Necrolysis (TEN) patients receiving either systemic IVIG or Ciclosporin (CsA) as initial treatments.

**Methods:**

Retrospective review of consecutive patients admitted for SJS/TEN at the Singapore General Hospital (SGH) from 2011 to 2017 who received either IVIG or Ciclosporin at the onset of the disease and had ophthalmological follow-up of at least 6 months were included. Acute ocular severity of SJS/TEN was graded using the Gregory grading score; chronic ocular complications were graded using the Sotozono system.

**Results:**

A total of 18 subjects were included for analysis, with eight in the IVIG group and 10 in the CsA group. There were no significant differences in acute Gregory severity grading between the two groups. The CsA group had a trend towards worse overall chronic Sotozono grading scores compared to the IVIG group (median [IQR]: 2 [0–3] vs. 1 [0–6.5], *p* = 0.27), with a higher incidence of acute severe cornea involvement (60% vs. 25%, *p* = 0.93) and chronic corneal and eyelid involvement in the former than the latter. SJS/TEN patients with worse acute ocular involvement were more likely to have TEN and perianal mucosal involvement (50% vs. 0, *p* = 0.01).

**Conclusion:**

Compared to those who received IVIG, SJS/TEN patients who received CsA at the acute disease stage, seemed to have worse acute corneal and chronic corneal and eyelid complications. Larger studies are needed to confirm this finding.

## Introduction

Stevens-Johnson syndrome (SJS) and its more severe variant, toxic epidermal necrolysis (TEN), are acute self-limiting diseases of the skin and mucous membranes with potential life-threatening complications (1). Ocular complications which range from mild to severe in SJS/TEN occur acutely in 50–88% of patients (2–4), with close to 80% having long-term ocular complications (5). Chronic ocular sequelae includes two broad categories: limbal stem cell failure, such as conjunctivalisation, corneal neovascularisation, and persistent epithelial defects, and conjunctival failure including goblet cell deficiency, dry eyes, forniceal shortening and symblepharon (6). Involvement of the eye lids and other ocular adnexa, such as trichiasis, lid-margin scarring, eyelid malpositions and meibomian gland dysfunction, are risk factors that exacerbate the ophthalmic sequalae of SJS/TEN. Corneal scarring can cause permanent visual loss and chronic inflammation can cause ocular discomfort, resulting in significant ocular morbidity for SJS/TEN patients with severe disease (7).

There are various proposed immune-mediated associations between the development of SJS/TEN with certain drugs, infections and ethnicities (8). The hallmark of SJS/TEN is epidermal cell apoptosis mediated through keratinocyte Fas–FasL interaction or T-cell release of perforin and granzyme B (9). The current standard of care for SJS/TEN is largely supportive; recently various immunomodulating therapies have been introduced which include systemic corticosteroids, intravenous immunoglobulins (IVIG), cyclosporin A (CsA), anti-tumour necrosis factor biologics, and plasmapheresis (10). To date however, there is still a lack of definitive evidence to demonstrate clear benefit of any one intervention over the other still, with further multicentre, randomised studies still needed to confirm these findings (11, 12).

The use of systemic CsA has been shown in some studies to reduce the mortality rate in SJS/TEN (13–16), CsA is a polypeptide that inhibits CD8 activation, and is hypothesised to inhibit downstream cytotoxic epidermal apoptosis through either FasL or the perforin-granzyme pathway (17). On the other hand, IVIG, a heterogenous pooled plasma of donor human IgG, has been shown to reduce healing time and hospitalisation duration in SJS/TEN (18, 19). The basis for the use of IVIG in SJS/TEN is via a combination of many pathways, including interfering with T cells, B cells, and monocytes which involve blocking the interaction of Fas with FasL (20–22). However the evidence for the benefit of either CsA or IVIG is conflicting and there remains no consensus on definitive dosages or duration of treatment in SJS/TEN (11, 23).

Few studies have evaluated the effects of systemic CsA or IVIG on acute and chronic ocular SJS/TEN outcomes. The existing studies could not identify therapeutic benefits of initial IVIG (24, 25) or systemic CsA (26) given in the acute phase on final visual outcomes and chronic ocular outcomes. Thus, our study aims to compare the acute and chronic ocular complications of patients who received either systemic CsA or IVIG during the acute stages of SJS/TEN.

## Methods

Medical records of patients admitted to the Singapore General Hospital with a diagnosis of SJS, TEN, or overlap SJS/TEN and referred to the Ophthalmology department from January 2011 to December 2017 were retrospectively reviewed. We included patients with an ophthalmological follow-up period of at least 6 months (i.e., excluded those who passed away during the acute hospital admission period). We also excluded patients who were treated only with supportive care, who received other agents such as systemic corticosteroids and those with a past history of corneal or conjunctival surgery. The research adhered to the tenets of the Declaration of Helsinki and ethics committee approval was obtained from the Institutional Review Board of Singhealth (IRB number 2014/2011). Written informed consent was obtained from all subjects.

The diagnosis of SJS, TEN or SJS/TEN was made by a dermatologist in the acute disease phase based on the clinical features of epidermal detachment and mucositis with supportive histology (16). An algorithm of drug causality for epidermal necrolysis (ALDEN) was used to assess for drug causality. SJS and TEN were characterised by an epidermal body surface area (BSA) detachment of less than 10% and more than 30% of the BSA, respectively. SJS/TEN overlap has 10–30% detachment (27). Clinical data on the following were collected: patient demographics, past medical history, inciting cause for SJS/TEN, mucocutaneous involvement of oral, genital or perianal sites, SCORTEN, and length of hospital stay. SCORTEN is a prognostication score specifically for SJS and TEN patients that is calculated on arrival and provides an accurate estimation of the risk of death, with one point given for each of seven clinical and laboratory variables met, namely age >40 years old, malignancy, heart rate >120 beats per minute, BSA detachment >10%, serum urea >10 mmol/L, serum glucose >14 mmol/L and bicarbonate. The probability of mortality predicted by the score is 3.2% for 0–1 point, 12.1% for 2, 35.3% for 3, 58.3 for 4 and 90% for ≥5 points (28). For the purpose of this study, the initial treatment regimen of either IVIG or oral CsA administered within the first 7 days of admission and the need for long-term follow-up and systemic immunosuppression were recorded.

All patients with SJS/TEN or overlap were considered for oral CsA or IVIG therapy if they met the following criteria (1) age older than 18 years old, (2) admission no later than 7 days after the onset of blistering, and (3) progressive disease activity. Patients were excluded if they had (1) renal impairment, unless they were on long-term renal replacement therapy, (2) uncontrolled hypertension, (3) severe infection, (4) active malignancy, (5) HIV infection, or (6) were pregnant. For patients recruited before 2012 as part of the institution’s standardised treatment protocol based on prevailing evidence, all patients were given IVIG as follows: 0.5 mg/kg over 4 days or 1 g/kg over 3 days (29, 30). From 2012 onwards again as part of a change in the institution’s standardised treatment protocol due to suggestion that IVIG had no survival benefits in our local cohort (16), all patients were then standardised to be given oral or via nasogastric tube CsA based on a published oral regimen from the French referral centre for toxic bullous diseases (16) as follows: 3 mg/kg/day for 10 days, followed by 2 mg/kg/day for 10 days then lastly 1 mg/kg/day for 10 days. All patients were given treatment within the first 1 week of their admission and completed their treatment regimen. No other systemic immunosuppression was administered during the treatment of their disease.

### Acute phase ocular grading

The data from the worse eye was recorded for the study. A detailed ophthalmic examination was performed by the referring ophthalmologist once the diagnosis of SJS/TEN had been made within 24 h of admission. Ophthalmic data collected included best corrected visual acuity (BCVA), tonometry, and a slit-lamp examination with fluorescein staining. Ocular surface findings during the acute phase, defined as findings at the first ophthalmological consultation, were graded by a published acute ocular SJS grading by Gregory et al. based on the extent and location of epithelial sloughing as mild, moderate, severe or very severe (31). Mild cases had only conjunctival hyperaemia with no epithelial sloughing in any areas of the ocular surface or lid margins. Moderate cases had no corneal sloughing beyond punctate keratopathy, but did have limited, discrete sloughing of the conjunctiva with bulbar or palpebral conjunctival staining that measured less than 1 cm in largest diameter (areas of staining significantly smaller than the areas without staining or epithelial sloughing) or lid margin sloughing or staining including less than one-third of the length of the lid margin. Severe case had at least 1 of the following staining criteria: (1) corneal epithelial defect beyond punctate keratopathy, (2) at least 1 lid margin with staining involving more than one-third of its length, and (3) any section of bulbar or palpebral conjunctiva with staining of more than 1 cm in largest diameter. To qualify as severe, only 1 of these 3 criteria had to be met, not all 3 at once. Extremely severe cases were those who simultaneously met the severe criteria on more than 1 lid margin and on multiple sections of the bulbar and palpebral conjunctiva (Supplementary Table 1). Topical eyedrops and acute intervention administered such as an amniotic membrane transplant (AMT) at the acute and chronic phase (defined as duration of 6 months or longer) were also recorded, which were left to the treating ophthalmologist’s discretion.

### Chronic phase grading

Patients who had 6 months or longer follow-up visit had a similar ocular data set collected at their latest visit. Chronic ocular involvement was graded based on the chronic ocular surface complications score (COCS) by Sotozono et al. for corneal, conjunctival and eyelid complications (32). The 13 factors included in the score are shown in Supplementary Table 2, with each component graded on a scale of 0–3 depending on severity as described.

### Statistical analysis

All data analyses were performed using SPSS version 28 (SPSS, Inc., Chicago, IL, USA). Continuous values were expressed as median (interquartile range [IQR] 25–75 percentile), categorical variables are presented as frequencies in percentages. Outcomes were compared between patients who received CsA vs. those who received IVIG in the acute disease stage. We also assessed the relationship of acute Gregory ocular score and COCS with the variables. We defined the anticipated effect size of 1.70-fold between the groups to be clinically significant. Thus, an estimated sample size of eight eyes in each group will be needed to give the study 80% power to show a difference between the groups, with a statistical precision of no worse than 0.05. A secondary analysis was carried out on the same 18 subjects to evaluate for variables associated with the severity of acute or chronic ocular involvement. Mann–Whitney U test was used for continuous variables, and McNemar Chi-square test for categorical variables for analysis. Statistical significance was defined as *p* < 0.05.

## Results

Eighteen patients with SJS/TEN who met our inclusion criteria were recruited into the study. The baseline characteristics of patients in both groups and further details of the acute SJS/TEN episode are in Table 1. The mean age of the study cohort was 43 (31–69) years old with 15 (83.3%) males, and the majority being Chinese (*n* = 16, 88.9%). 7 (38.9%) had SJS, 7 (38.9%) had TEN, and 4 (22.2%) had overlap SJS/TEN disease. There were eight patients in the IVIG group and 10 patients in the CsA group. The most common inciting causes for SJS/TEN in our cohort was allopurinol (*n* = 4), antibiotics (*n* = 4) (co-trimoxazole [*n* = 1], amoxicillin [*n* = 3]), non-steroidal anti-inflammatory drugs (*n* = 4), anti-epileptic medications (phenytoin, carbamazepine, lamotrigine, *n* = 1 each) and omeprazole (*n* = 1). Two patients were noted to have other causes related to possible traditional Chinese and Malay medicinal use, respectively.

**Table 1:** Characteristics between those who received IVIG vs. Ciclosporin for SJS/TEN at the acute onset.

Characteristics	Median (IQR)/*n* (%)		
	IVIG (*n* = 8)	Ciclosporin (*n* = 10)	*p*-value
Age, median (IQR)	47.0 (43–71)	34.0 (29.5–64.8)	0.12
Male	5 (62.5)	10 (100.0)	0.07
Race, number (%)			0.42
Chinese	8 (100.0)	8 (80.0)	
Malay	0	1 (10.0)	
Others	0	1 (10.0)	
Disease classification (%)			0.33
SJS	1 (13.5)	6 (60.0)	
TEN	4 (50.0)	3 (30.0)	
Overlap SJS/TEN	3 (37.5)	1 (10.0)	
Cause of SJS/TEN (%)			0.25
Non-steroidal anti-inflammatories	2 (25.0)	2 (20.0)	
Idiopathic	1 (12.5)	1 (10.0)	
Allopurinol	1 (12.5)	3 (30.0)	
Anti-epileptics	2 (25.0)	1 (10.0)	
Antibiotics	1 (12.5)	3 (30.0)	
Omeprazole	1 (12.5)	0	
SCORTEN, median (IQR)	3 (2–3)	1 (1–2)	0.11
Maximal BSA detached %, median (IQR)	30 (15–90)	45 (22.5–83.8)	0.12
Associated mucosal involvement, (%)			
Oral	6 (75.0)	10 (100.0)	0.13
Genital	5 (62.5)	6 (60.0)	0.35
Perianal	3 (37.5)	4 (40.0)	0.64
Length of hospital stay, days, median (IQR)	17 (13–43)	14 (8.3–24.8)	0.60
Gregory grading (*n*)			0.62
Mild	2 (25.0)	1 (10.0)	
Moderate	0	1 (10.0)	
Severe	2 (25.0)	4 (40.0)	
Very severe	4 (50.0)	4 (40.0)	
Cornea involvement (*n*)			0.93
None-mild	5 (62.5)	3 (30.0)	
Moderate	1 (12.5)	1 (10.0)	
Severe	0	3 (30.0)	
Very severe	2 (25.0)	3 (30.0)	
Conjunctival involvement (*n*)			0.51
None-mild	2 (25.0)	6 (60.0)	
Moderate	2 (25.0)	1 (10.0)	
Severe	1 (12.5)	1 (10.0)	
Very severe	3 (37.5)	2 (20.0)	
Lid involvement (*n*)			0.51
None-mild	2 (25.0)	6 (60.0)	
Moderate	2 (25.0)	1 (10.0)	
Severe	1 (12.5)	1 (10.0)	
Very severe	3 (37.5)	2 (20.0)	
Use of topical drops, (*n*)	7 (87.5)	7 (70.0)	0.57
Types of drops (*n*)			0.41
Dexamethasone	6 (75.0)	6 (60.0)	
Pred forte	1 (12.5)	3 (30.0)	
Amniotic membrane transplant (*n*)	0	1 (10.0)	0.90

At their initial presentation, there were no other significant differences between the two groups with respect to age, gender, race, disease classification, length of entire hospital stay, SCORTEN grade, and acute ocular involvement (*p* > 0.05 for all). However, we noted a higher incidence, though not significant, of acute severe or worse corneal involvement (60% vs. 25%, *p* = 0.93) in the CsA compared to the IVIG group. The most prescribed eye drop was dexamethasone in both groups. One patient (10%) in the CsA group required an AMT, whereas none in the IVIG group required an AMT (Table 1).

In terms of long-term follow-up duration, there was no significant difference noted between both CsA and IVIG groups (19.5 [11–54] vs. 17 [12–60] months., *p* = 0.88) (Table 2). In the CsA group, 70% still required ongoing specialist follow-up, with 6 out of 7 (85.8%) having a poor ocular surface needing punctal plugs or topical CsA 0.5% eye drops, and 4 (57.1%) having eyelid complications including trichiasis, distichiasis, cicatricial entropion and symblepharon. Two of the patients with a poor ocular surface went on to have recurrent corneal erosions and persistent epithelial defects 3 years after her initial presentation. In the IVIG group, one patient (12.5%) required ongoing ophthalmic follow-up due to distichiasis and symblepharon that compromised the ocular surface. The overall COCS score (2 [0–3] vs. 1 [6–6.5], *p* = 0.27), proportions of those with chronic cornea and eyelid complications, and those requiring long-term topical steroids were also greater in the CsA group compared to the IVIG group, although these differences were not significant between the two treatment groups in view of a small sample size. Two patients in the CsA group had to undergo further ocular surgical interventions, including 1 who needed AMT and the other who needed multiple therapeutic cornea grafts due to repeated failures and eventually needing enucleation secondary to a painful blind eye. No patients in the IVIG grouped required long-term surgical interventions. There were no differences between the two groups in terms of the need for long-term systemic immunosuppression.

**Table 2:** Chronic ocular complications in IVIG vs. Ciclosporin treatment groups.

Characteristics	Median (IQR)/*n* (%)		
	IVIG (*n* = 8)	Ciclosporin (*n* = 10)	*p*-value
Duration of follow-up time point (months), median (IQR)	19.5 (11–54)	17 (12–60)	0.88
Sonotozo grading score (*n*)	2 (0–3)	1 (0–6.5)	0.27
Corneal complications			
SPK	0.63 (0.74)	1.11 (1.27)	0.36
Epithelial defect	0	0.56 (1.13)	0.18
Loss of POV	0	0.33 (1.0)	0.36
Conjunctivalisation	0	0.89 (1.17)	0.05
Neovascularisation	0.25 (0.71)	0.67 (1.12)	0.38
Opacification	0.13 (0.36)	0.44 (1.01)	0.41
Keratinisation	0	0.44 (1.01)	0.24
Conjunctivalisation complications			
Hyperaemia	0	0.22 (0.44)	0.17
Symblepharon formation	0.63 (0.92)	0.22 (0.44)	0.26
Eyelid complications			
Trichiasis	0.25 (0.71)	0.33 (0.50)	0.78
Mucocutaneous junction involvement	0.63 (1.19)	0.67 (0.87)	0.94
Meibomian gland involvement	0.25 (0.46)	0.44 (0.53)	0.43
Punctal gland involvement	0	0	
Number of eyes with complications (*n*)			0.28
Persistent epithelial defect	0	2 (20.0)	
Moderate- severe dryness	0	6 (60.0)	
Trichiasis/distichiasis	1 (12.5)	2 (20.0)	
Entropion	0	1 (10.0)	
Symblepharon	1 (12.5)	1 (10.0)	
Topical steroid drops (*n*)	1 (12.5)	5 (50.0)	0.13
Dexamethasone	1 (12.5)	3 (30.0)	
Pred forte	0	1 (10.0)	
Betamethasone	0	1 (10.0)	
Need for surgical intervention (*n*)	0	2 (20.0)	0.05
AMT	0	1 (10.0)	
Cornea graft	0	1 (10.0)	

A secondary analysis was carried out to evaluate for variables associated with the severity of acute or chronic SJS/TEN ocular involvement. Patients with a worse acute Gregory score were more likely to have TEN (50% vs. 0% in severe vs. mild ocular involvement, *p* = 0.01) and have perianal mucosal involvement (85.7% vs. 0% severe vs. mild ocular involvement, *p* = 0.01) than those with a better Gregory score. Patients with high COCS were also more likely to have severe acute corneal involvement than those with low COCS (80% vs. 7.8% in high vs. low COCS groups, *p* = 0.01). There were no other significant differences in their baseline characteristics or other variables between the two groups at the acute or chronic phase, such as percentage of maximal BSA detached, type of disease (SJS/TEN), or SCORTEN (Results not shown).

## Discussion

Earlier evidence showed that systemic CsA (13–16) and IVIG (18–21) may reduce mortality rates in SJS/TEN. However much fewer studies have evaluated the effect of systemic CsA or IVIG given in the acute phase on acute or chronic ocular outcomes. In our study of 18 subjects, there were no significant differences in acute ocular or systemic involvement between those who received systemic CsA or IVIG. Subjects who received systemic CsA however seemed to have a higher incidence of acute corneal and chronic corneal and eyelid complications compared to those who received IVIG. Larger studies are needed to confirm this finding.

From our results, we noted that in the acute phase, although not statistically significant, the severity of corneal involvement differed between the two groups. In the IVIG group, corneal involvement was none to mild in five cases (62.5%) and very severe in two cases (25%), while in the CsA group it was none to mild in three cases (30%) and severe to very-severe in six cases (60%). It is possible that this difference in the severity of the corneal involvement may have affected the ocular sequelae at the chronic phase. However the number of patients in this study was too small to make a statistically significant comparison of the ocular surface severity at the acute phase.

In the chronic phase of disease, there was a statistically insignificant trend towards a greater number of patients who received systemic CsA with chronic corneal and eyelid complications on follow-up compared to those who received IVIG. These were mainly due to persistent epithelial defects, moderate to severe ocular surface dryness, and eyelid malpositions such as trichiasis/distichiasis, entropion or symblepharon. Two subjects in the CsA group required ocular intervention in the form of 1 AMT and PK each, compared to none in the IVIG group. These findings suggest that those in the CsA group who had more severe acute cornea involvement had worse chronic ocular morbidity than the IVIG group. This is also reflected by our findings that more severe acute corneal involvement was associated with worse chronic ocular sequelae, consistent with a previous study in which the extent of acute ocular involvement was found to be a significant risk factor for poor vision of BCVA <20/200 in the chronic phase (33). Other studies have also demonstrated that eyes with worse acute cornea involvement were more likely to have more severe chronic ocular sequelae (23, 34, 35). Limbal stem cell deficiency during the acute phase leads to persistent epithelial defects, corneal conjunctivalisation, keratinisation and opacification that result in devastating long-term visual morbidity and sequalae. This suggests that subjects with more severe corneal involvement in the acute phase are higher-risk for chronic ocular sequelae in SJS/TEN, and would warrant closer monitoring and more intensive and earlier intervention to reduce visual morbidity.

Our study results also suggest that patients with worse acute ocular involvement were more likely to have TEN and perianal mucosal involvement than those with milder ocular involvement. Similarly, earlier studies reported higher proportions of TEN patients with acute ocular surface inflammation compared to SJS patients, although not statistically significant (36, 37). It has been deduced that more diffuse systemic mucous and cutaneous damage in TEN compared to SJS may be associated with a higher risk of damage to the ocular surface (36). Similar to earlier studies, we also observed that other presenting features of systemic disease severity at the onset were not a good guide to predicting late ocular complications, such as acute SCORTEN (28). The SCORTEN includes clinical parameters that reflect the patients’ systemic condition to predict disease severity and morbidity. As the ocular surface has a privileged immune response (28), there might be a discordance between the ocular and systemic manifestations of the disease.

Systemic CsA has been regarded with a potential to reduce overall mortality in SJS/TEN patients in both adult and paediatric populations (13–16). Interestingly, our data from a limited cohort did not show a clear advantage of CsA over IVIG on acute and chronic ocular involvement. Hall et al. also reported no association of systemic CsA used initially with reduced chronic ocular complications in a small single-centre comparative cohort study of 28 eyes from 14 SJS/TEN patients (26).

Regarding the effects of systemic IVIG on ocular SJS outcomes, Kim et al. observed that IVIG significantly improved in visual acuity and ocular involvement, especially if administered within 6 days of the disease onset (24). On the contrary, Yip et al. reported that IVIG was not associated with reduced severity of ocular complications in patients with SJS/TEN (25). However, this study was limited by a small number of 10 patients with only one patient treated with IVIG alone, while other patients were treated with IVIG either before or after steroid treatment that could confound the results. Other studies have not supported the use of IVIG alone for SJS/TEN as there were no clear benefits in reducing ocular disease progression or mortality rates (38, 39). Previous studies evaluating the systemic benefits of IVIG in combination with corticosteroids have however shown stronger evidence in reducing the recovery time and mortality risks in SJS/TEN, especially in Asians (18, 19). A multicentre, single-arm study of Japanese adult SJS/TEN patients who did not respond sufficiently to systemic steroids reported that additional IVIG administered early at 400 mg/kg/day for 5 days showed improved ophthalmic and skin lesions in six out of seven patients (40). The investigation of pulsed systemic steroids combined with either IVIG or CsA is being planned in our future work.

The advantage of IVIG over systemic CsA in reducing chronic ocular sequelae in SJS/TEN is controversial. Murata et al. hypothesised in SJS/TEN, FasL levels increase several days before clinical manifestations appear, and decrease rapidly to reach the normal range 5 days after disease onset (41). Thus, early IVIG treatment may provide better therapeutic effect by saturating the Fas binding site. In the ocular surface, the FasL is highly expressed in the cornea and Fas–FasL interactions may contribute to chronic inflammation through FADD-MyD88 interactions (42). The inhibition of Fas–FasL interaction and its downstream inflammatory responses mediated by IVIG could dampen chronic ocular inflammation to a greater extent, compared to that of CsA which downregulates activated T cells and inflammatory cytokines such as IL-6 and MMP-9 in the ocular surface; but further work is required to prove such a claim.

Earlier meta-analyses have described mixed results on the benefits of biologic TNF-α inhibitors such as etanercept and infliximab in SJS/TEN (43, 44), with the major concern being evidence based only on small cohorts. However, none of these studies have so far reported the impact of these biologics on ocular outcomes. More robust studies would be needed to confirm the efficacy of these drugs in systemic and ocular SJS/TEN.

Based on our study findings, the risk of SJS/TEN and the associations between various causative drugs is significant enough to warrant investigating potential underlying potential predispositions to severe ocular complications. Existing studies indicate a genetic predisposition to certain drugs in various population groups (45, 46), and future research should continue to explore these genetic links.

Limitations of our study include the retrospective design and small sample size, which is expected given the low incidence of the disease. The study’s sample size may be underpowered to detect significant differences between groups. Nonetheless, we hope that our results can add to the currently limited literature on systemic immunomodulation in ocular SJS/TEN and perhaps encourage future larger trials to confirm or challenge our findings. Clinical data were recorded by various on-duty ophthalmologists although all with similar levels of clinical training and reflects real-world data. There may be a therapeutic window which has yet to be defined during which the administration of CsA or IVIG may have positive ocular outcomes. In some literature, 3–6 mg/kg body weight per day of CsA is recommended, but in our study, 3 mg/kg body weight per day was started for 10 days, followed by 2 mg/kg body weight per day for 10 days and then tapered. Perhaps the initial dosage administered may have been inadequate to effectively reduce systemic and ocular inflammation. We also realise that there could be other confounding factors affecting our results. Ocular treatment in the acute phase was not standardised due to variations amongst treatment regimens of various ophthalmologists, such as type of steroid eyedrops received, or how soon AMT was performed if necessary. We found a low incidence of AMT performed in our cohort, where its use in the acute phase was only standardised more recently in our institution. This may have a bearing of long-term ocular outcomes and prognosis. The exact number of days after admission prior to administration of the treatment was not recorded for each subject. Despite these limitations, this is the first study that evaluated the differences in acute and chronic ocular outcomes between systemic CsA and IVIG in SJS/TEN patients. The administration protocols of CsA and IVIG were standardised and no patient received additional systemic immunosuppression other than their initial drug. The findings of our study would still require further validation with large cohort studies.

In conclusion, our study suggests that compared to those who received IVIG, SJS/TEN patients who received CsA at the acute disease stage seemed to have worse acute corneal and chronic corneal and eyelid complications. Patients with worse chronic ocular morbidity were also more likely to have more severe acute corneal involvement. SJS/TEN patients with worse acute ocular involvement were more likely to have TEN and perianal mucosal involvement. Further and larger studies are required to validate these findings. More effective therapeutic regimens need to be explored in mitigating acute and chronic ocular complications in SJS/TEN, such as the use of biologics or combination therapies of pulsed steroids with other immunosuppressives.

## Data availability statement

The original contributions presented in the study are included in the article. Further inquiries can be directed to the corresponding author.

## Ethics statement

The studies involving humans were approved by the Singhealth Institutional Review Board. The studies were conducted in accordance with the local legislation and institutional requirements. Written informed consent for participation was not required from the participants or the participants’ legal guardians/next of kin in accordance with the national legislation and institutional requirements.

## Author contributions

VF: Conceptualization, Data curation, Formal analysis, Investigation, Methodology, Project administration, Resources, Software, Writing – original draft, Writing – review & editing. LY: Conceptualization, Data curation, Investigation, Methodology, Resources, Validation, Writing – review & editing. JM: Investigation, Methodology, Project administration, Resources, Supervision, Writing – review & editing. HO: Conceptualization, Data curation, Formal analysis, Investigation, Methodology, Resources, Supervision, Validation, Writing – original draft, Writing – review & editing.
