# Supplementary material for: Acute and chronic ocular outcomes in SJS/TEN patients treated with oral ciclosporin vs intravenous immunoglobulin
Source: Front Med (Lausanne). 2024 Aug 19;11:1398506. doi: 10.3389/fmed.2024.1398506 (PMC11368074; doi:10.3389/fmed.2024.1398506)
Supplement: Supplementary file 1 [file Table_1.docx]

**Supplementary Table 1. Acute Ocular SJS Grading Criteria^34^**

| **Staining Location** | **Severity of Eye Involvement** | | | |
| --- | --- | --- | --- | --- |
|  | **Mild** | **Moderate** | **Severe** | **Very Severe** |
| Lid Margin | No stain | Stain <1/3 of lid margin length | Stain > 1/3 of lid margin length on at least 1 lid | Stain >1/3 of lid margin length on more than 1 lid |
| Cornea | No stain | No stain | Any epithelial defect more than punctate staining | Any epithelial defect mor than punctate staining |
| Conjunctiva (bulbar and palpebral) | Hyperaemia, without staining | (+) Stain, <1cm in greatest diameter | (+) Stain > 1cm | Multiple areas of stain >1cm |

**Supplementary Table 2. Sotozono grading system for chronic ocular SJS manifestations^13^**

|  | **None (0)** | **Mild (1)** | **Moderate (2)** | **Severe (3)** |
| --- | --- | --- | --- | --- |
| Cornea complications |  |  |  |  |
| Superficial Punctate Keratopathy | No staining | Sparse | Moderate | High |
| Epithelial defect | Absent | <1/4 of cornea surface | ¼ to ½ of cornea surface | >1/2 of cornea surface |
| Loss of Palisades of Vogt | Presence of entire POV | Loss of <1/2 of entire circumference of POV | Loss of > ½ of entire circumference of POV | Total loss of POV |
| Conjunctivalisation | Absent | <1/4 of cornea surface | ¼ to ½ of cornea surface | >1/2 of cornea surface |
| Corneal Neovascularisation | Absent | Neovascularisation confined to corneal periphery | Neovascularisation extending up to pupil margin | Neovascularisation extending beyond pupil margin into central cornea |
| Corneal Keratinisation | Absent | <1/4 of cornea surface involved | ¼ to ½ of cornea surface | >1/2 of cornea surface involved |
| Conjunctival complications |  |  |  |  |
| Hyperaemia | Absent | Mild | Moderate | Severe |
| Symblepharon | Absent | Symblepharon formation involving only conjunctival surface | Symblepharon formation involving <1/2 of cornea surface | Symblepharon involving >1/2 of cornea surface |
| Eyelid Complications |  |  |  |  |
| Trichiasis | Absent | Involving <1/4 of lid margin | Involving ¼ to ½ of lid margin | Involving >1/2 of lid margin |
| Mucocutaneous Junction Involvement | Normal | Mild irregularity | Moderate irregularity | Severe irregularity |
| Meibomian Gland Involvement | Clear oily fluid expressed | Yellowish-white oily fluid expressed | Thick cheesy material expressed | Inability to express any fluid from the meibomian glands |
| Punctal Involvement | Normal patent puncta | Iatrogenic punctal occlusion (plugs or suture) | Superior or inferior puncta occluded by scarring | Both superior and inferior puncta occluded by scarring |
